# Oral sodium hyaluronate relieves knee discomfort: A 12‑week double‑blinded, placebo‑controlled study

**DOI:** 10.3892/etm.2023.12352

**Published:** 2023-12-15

**Authors:** Kiichi Sugiyama, Mariko Oe, Tomomi Tanaka, Ryosuke Matsuoka, Yumi Takeda, Mamoru Kimura, Koji Odani

**Affiliations:** 1 Personal Coaching Laboratory, Hokkaido University of Education, Iwamizawa, Hokkaido 068-0835, Japan; 2 Research and Development Division, Kewpie Corporation, Tokyo 182-0002, Japan; 3 Sapporo Columbia Medical Office, Sapporo, Hokkaido 060-0001, Japan

**Keywords:** hyaluronic acid, hyaluronan, dietary supplement, knee, joint

## Abstract

Sodium hyaluronate (SH) is a high molecular-weight polysaccharide composed of repeating polymeric disaccharides of D-glucuronic acid and N-acetyl-D-glucosamine. SH is present in every connective tissue and organ, with synovial fluid having the highest concentration of SH in the body. The effectiveness of oral SH on gonarthrosis is known; although, its influence on the knees of healthy individuals is not. However, as severe diseases may require surgery, it is better to take care of healthy knees before the onset of gonarthrosis. Therefore, the present study investigated the functionality of SH on the knee of healthy individuals. The present study was a randomized double-blind, placebo-controlled trial in which healthy adults (mean age: Placebo group, 61.50±1.59; SH group, 58.50±1.81), rated as grade ≤1 based on the Kellgren-Lawrence classification, were administered 111 mg/day SH for 12 weeks. The evaluation of visual analog scales were performed to assess the discomfort in the knees of the participants and were conducted at baseline, and then 6 and 12 weeks after the start of SH ingestion. Additionally, a locomotive syndrome risk test quantifying the mobility of the participants, a one-leg standing time with eyes open test evaluating the strength of the leg muscle as well as the ability to balance by measuring the time to stand on one leg and a blood test (interleukin-10, aspartate aminotransferase, alanine transaminase, γ-glutamyl transferase, lactate dehydrogenase, creatine kinase and C-reactive protein) were performed at baseline and then 12 weeks after the start of SH ingestion. A significant suppression of knee symptoms were demonstrated in the SH group compared with the placebo group in terms of the total visual analog scale scores for pain, stiffness and discomfort for the 31 healthy adult subjects. Significant suppression of symptoms was also demonstrated in the placebo group in terms of discomfort in the knees when descending stairs and pain in the knees after walking for a longer distance or duration than normal. No significant differences between the two groups were demonstrated in the locomotive syndrome risk test, one-leg standing time with eyes open test and the blood test. The results of the present study suggest the possibility that oral SH may help to maintain a healthy condition of the knees. The study protocol was registered with the University Hospital Medical Information Network Clinical Trial Registry in advance (registration no. UMIN000045980, November 4, 2021).

## Introduction

Musculoskeletal disorders are on the rise worldwide due to the aging and growing population (1). In particular, osteoarthritis (OA) is a major cause of health problems globally, and worldwide estimates suggest that 250 million individuals are affected, and is the main reason for functional decline and pain of the knee (2). It is particularly prevalent in developed countries where a greater number of individuals are reaching an advanced age, and it was estimated that the number of patients with OA in the US would exceed 60 million in 2020(3). OA is caused by the gradual wear and tear of cartilage over numerous decades, and the accompanying pain reduces quality of life (4). Severe cases of OA can be treated with knee osteotomy or knee replacement, and sodium hyaluronate (SH), paracetamol, nonsteroidal anti-inflammatory drugs or corticosteroids can be administered by intraarticular injection as a conservative treatment. However, surgery or regular hospital visits can be a burden on patients (5,6). Control of body weight with appropriate exercise, a balanced diet and care for the knees using dietary supplementation (such as glucosamine or chondroitin) are recommended to prevent severe knee discomfort (7-9).

In Japan, ~10 million individuals perform annual runs and ~3 million run at least twice a week. Furthermore, 250,000 have been reported to run full-length marathons at least once a year (10). However, numerous runners who are middle-aged and elderly experience issues centered around knee joints, such as gonarthrosis, due to physical decline from aging and overwork (11). To prevent the progression of running disorders, such as gonarthrosis, it is desirable to treat them as easily as possible at a relatively mild stage, such as when the affected individuals feel discomfort in the knees, before they become serious due to excessive training (12). Therefore, in the present study, the functionality of oral dietary supplements to alleviate knee pain and discomfort in healthy recreational runners who have yet to experience any running disorders was assessed.

Sodium hyaluronate (SH) is a high molecular-weight polysaccharide (~100-10,000 kDa) composed of repeating polymeric disaccharides of D-glucuronic acid and N-acetyl-D-glucosamine (13). SH is present in every connective tissue and organ with synovial fluid having the highest concentration of SH in the body at 3-4 mg/ml (14). There have been multiple studies on the effect of oral SH on gonarthrosis; however, most of them include severe cases such as patients with osteoarthritis of the knee, rated as grade ≥2 based on the Kellgren-Lawrence (KL) classification, and there are no reports of studies conducted only on healthy individuals (15-21). Therefore, a 12-week, randomized, double-blinded, placebo-controlled parallel study was performed, which recruited healthy adult men and women, rated as grade ≤1 based on the KL classification, meaning the individuals knees were healthy, with primary endpoints scored using visual analog scales (VAS) to evaluate the influence of oral SH on discomfort and pain in the knees (22).

## Materials and methods

### Study samples

In the present study, SH [Hyabest^®^ (S) LF-P; Kewpie Corp.] was used with dextrin as a placebo (Matsutani Chemical Industry Co., Ltd.), as dextrin is a safe food ingredient that has no effect on the knee (15,23). Both SH and the placebo (dextrin) were developed into capsule supplements by Aliment Industry Co., Ltd. and had the same taste and appearance. SH capsules had an SH content of 111 mg/capsule based on a qualitative analysis using high-performance liquid chromatography performed by the Japan Food Research Laboratories. The daily intake was one capsule.

### Study design and ethics

The present study was performed at Hokkaido University of Education (Hokkaido, Japan) between December 2021 and March 2022 under the administration of an internal medicine specialist. A total of 56 subjects, recruited specifically for the present study after obtaining written informed consent, were divided into two groups using block randomization to ensure there was no bias in sex or age (stratified randomization by sex and age). Grouping information was kept sealed and confidential from the time-point of grouping until the study was completed (24). The subjects, investigators, supplements provider and the outcome assessors were all blinded. The placebo group and the SH group both took one capsule per day over a period of 12 weeks. A VAS questionnaire regarding their knees was administered before the start of the study, at 6 weeks and at 12 weeks after starting treatment with the capsules. The duration of the study was chosen according to previous studies (25). A locomotive syndrome risk test (26-29), one-leg standing time with eyes open test (30), a blood test and physician interview were performed before the start of the study and at 12 weeks after starting treatment with the capsules. X-ray examination of the subjects' knees was performed during the study period in small groups and in no particular order at Hokkaido University Hospital (Hokkaido, Japan) under the administration of an orthopedic specialist. The present study complies with the Helsinki Declaration and the Ethical guidelines for Medical and Health Research Involving Human Subjects (Ministry of Education, Culture, Sports, Science and Technology; Ministry of Health, Labor and Welfare; and Ministry of Economy, Trade and Industry, Japan) (31) and was approved by Hokkaido University of Education Ethics Review Committee (approval no. 2021035001; March 26, 2021). The study protocol was registered with the University Hospital Medical Information Network Clinical Trial Registry in advance (registration no. UMIN000045980, November 4, 2021).

### Subjects

The subjects were 56 healthy adults (39 men and 17 women; mean age, 60.1±1.2 years). The inclusion criteria were as follows: i) Adult men and women aged 20-65 years; ii) determined to be healthy by the principal investigator of the present study, an internal medicine specialist, through blood tests assessing the levels of interleukin-10 (IL-10), aspartate aminotransferase (AST), alanine transaminase (ALT), γ-glutamyl transferase (γGTP), lactate dehydrogenase (LDH), creatine kinase (CK) and C-reactive protein (CRP), and medical interviews regarding the medical history of the individual; iii) experienced knee discomfort in the past year. The exclusion criteria were as follows: i) Taking medication or regularly going to the hospital; ii) a history of serious illness; iii) participation in another clinical trial within the past month; iv) habitual excessive eating or drinking, or irregular diet using a self-reporting method; v) deemed unsuitable by the screening physician (such as due to knee osteoarthritis or knee injury); vi) drug and food allergies; and vii) assessed as grade ≥2 based on KL classification, which indicates the individual has knee osteoarthritis. Participants were required to record their daily routines before (for 1 week within 1 month prior to intake) and during the study period; these were verified using diaries, which were used to confirm that there were no changes in lifestyle (such as diet and daily routines) before and during the study. The trial was explained in detail to the subjects and those who gave their written informed consent were enrolled in the study. The sample size was determined based on a similar previous study (32). As α=0.05 and power (1-β)=80%, the required sample size was ≥30. The Cancer Research and Biostatistics Statistical Tools (https://stattools.crab.org/) was used to calculate the sample size.

### VAS

A total of 21 items were selected from the Japanese Knee OA Measure (JKOM) and the Western Ontario and McMaster Universities OA Index that were suitable for the evaluation of healthy subjects (33,34). Knee pain, stiffness and discomfort were evaluated using VAS in the following situations: Upon awaking; climbing stairs; descending stairs; going to bed; bending and stretching the knee joint; walking for a longer distance or duration than normal; jogging for a longer distance or duration than normal. For VAS, 100-mm lines were used, with the left end of each line indicating the best condition without any symptoms and the right end indicating the worst condition ever experienced by the patient. Subjects evaluated their condition before they started taking the capsules, and at 6 and 12 weeks after they started taking them.

### Locomotive syndrome risk test

The locomotive syndrome risk test consisted of two physical examinations, the two-step test and the stand-up test, and the 25-question geriatric locomotive function scale (GLFS-25) questionnaire (26-29). The stand-up test evaluated whether the subjects could stand up from a sitting position in a chair at four different heights (40, 30, 20 and 10 cm) on one or both legs. The results of the stand-up test were scored as follows: 1, 10 cm on one leg; 2, 20 cm on one leg; 3, 30 cm on one leg; 4, 40 cm on one leg; 5, 10 cm on both legs; 6, 20 cm on both legs; 7, 30 cm on both legs; and 8, 40 cm on both legs. The scores were converted and used for analysis. A lower score on the stand-up test indicated a better result. The GLFS-25 were 25 questions that were answered on a 5-point Likert-type scale to assess the degree of locomotion. These tests were performed before the start of the supplement regimen and at 12 weeks after the start of the supplement regimen.

### One-leg standing time with eyes open test

The time a subject could stand on one leg with their eyes open (30) was measured using a digital stopwatch with a maximum length of 120 sec prior to the start of the supplement regimen and 12 weeks after the start of the regimen.

### Blood test

Blood samples from subjects were tested for levels of IL-10, AST, ALT, γGTP, LDH, CK and CRP prior to the start of the supplement regimen and at 12 weeks after the start of the regimen. The physician collected the blood samples, and the blood analysis was performed by Hoken Kagaku, Inc.

### Statistical analysis

Values are presented as the mean ± standard error and P<0.05 was considered to indicate a statistically significant difference. Statistical analysis was performed using SPSS Statistics 28.0 (IBM Corp.). Intergroup comparisons of changes in VAS were analyzed using two-way mixed analysis of covariance (ANCOVA) with initial values as covariates followed by a Sidak post-hoc test, and difference in the interaction effects, if significant. The intergroup comparisons applied ANCOVA with initial values as covariates, and comparisons before and after 6 and 12 weeks of the start of the supplement regimen were performed using a Sidak post-hoc test. Intergroup comparisons of the two-step test [two-step value=two-step length (cm)/height (cm)], one-leg standing time with eyes open test [duration of standing on one leg (sec)] and blood test were analyzed using two-way mixed ANOVA followed by a Sidak post-hoc test. Differences in the results of the stand-up test and GLFS-25 between the groups were assessed using the Mann-Whitney U-test with Bonferroni correction applied. In addition, before the start of the supplement regimen and at 12 weeks after the start of the regimen, the results were compared using the Wilcoxon signed-rank test with Bonferroni correction applied. Differences between groups for background characteristics such as age, height, body weight and body mass index (BMI) were assessed using the unpaired t-test and differences in the number of participants (men and women) between the groups were assessed using the Chi-squared test.

## Results

### Characteristics of subjects

**Figure 1:**
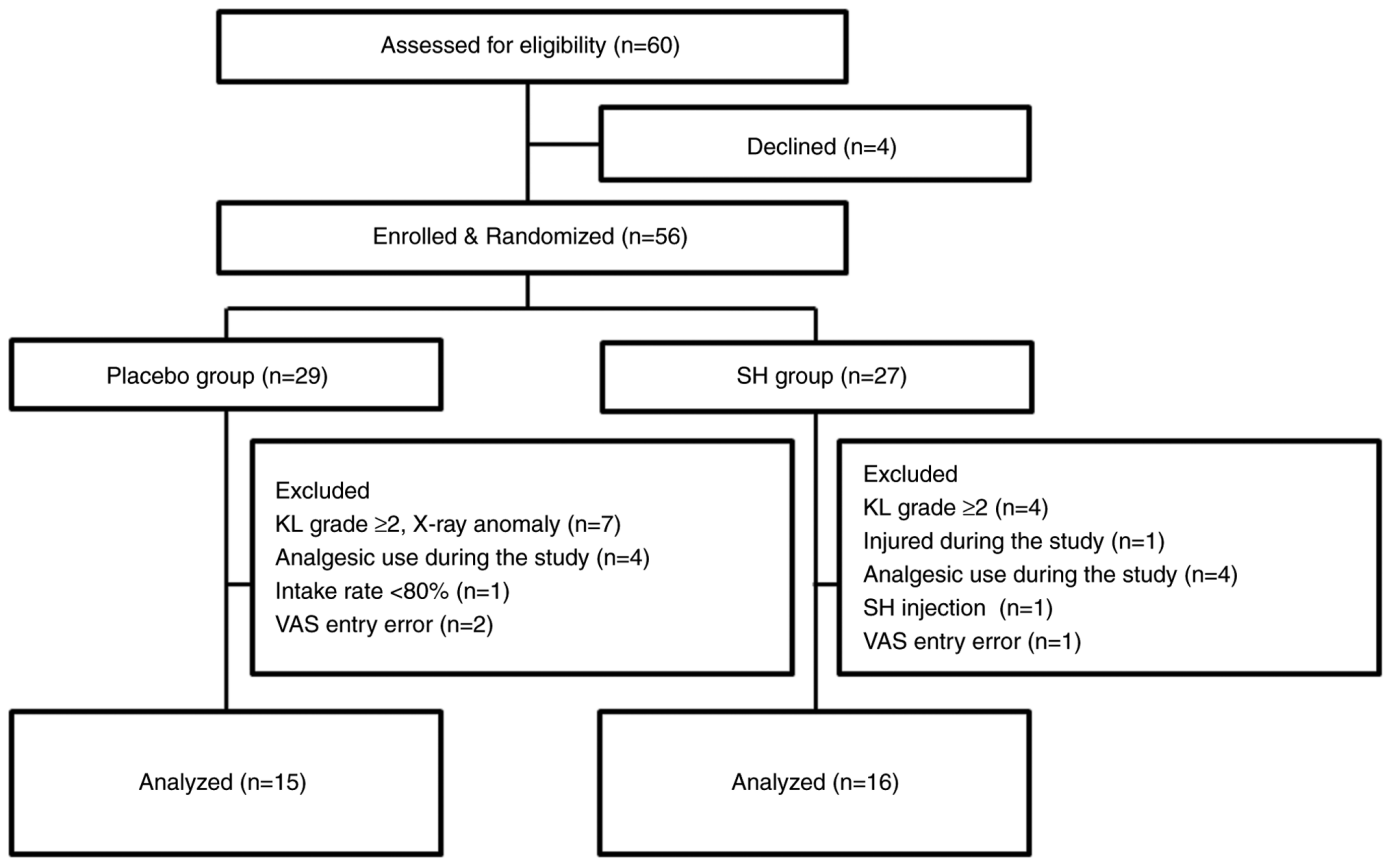
Flowchart of subject recruitment and assignment to the study groups. SH, sodium hyaluronate; KL, Kellgren-Lawrence classification; VAS, visual analog scale.

**Figure 2:**
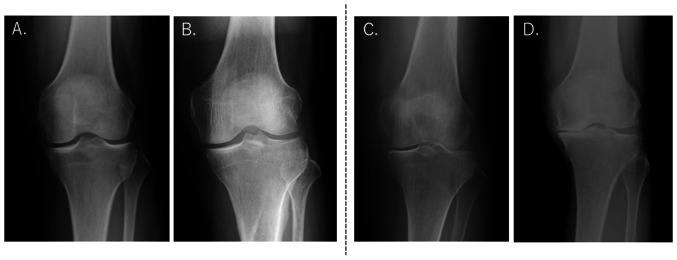
Representative X-ray images of knees from subjects who were (A) KL grade 0, (B) KL grade 1, (C) KL grade 2 and (D) KL grade 4. Selected for analysis were KL grades 0-1 and excluded from analysis were KL grades ≥2. KL, Kellgren-Lawrence classification.

Background characteristics of the study participants are presented in Table I. No significant differences in sex (placebo group, male:female=23:6; SH group, male:female ratio=16:11; P=0.103), age (placebo group, 61.50±1.59 years; SH group, 58.50±1.81 years), body height, weight and BMI were determined between the placebo and SH groups. None of the participants dropped out during the study period. Among the 56 study participants in the full analysis set, 25 were excluded who were found to have gonarthrosis of KL grade ≥2 based on knee X-rays or who had made errors when filling in the VAS. This resulted in a per protocol set consisting of 31 subjects for analysis (Figs. 1 and 2). The ingestion rate of the trial supplements was 98%. The diary records completed by the participants confirmed that the subjects included in the analysis did not change their diet or daily routine to a large extent during the trial period from the time before the trial, and they continued their training within the scope of daily life.

### VAS

Changes in VAS compared with the baseline are presented in Table II. Total scores for pain in the knees were significantly lower in the SH group than those in the placebo group at six weeks after the start of the supplement regimen and at 12 weeks after the start of the regimen (P<0.001). Total scores for knee pain significantly increased in the placebo group (P<0.001) when comparing the time before the start of the study and 12 weeks later; however, there was no significant difference in the SH group, which maintained a healthy condition. Total scores for knee stiffness in the SH group were significantly lower (P=0.017) at 12 weeks after the start of the regimen in comparison with the placebo group. The total score for knee stiffness significantly increased in the placebo group (P=0.030) before the regimen compared with after 12 weeks; however, there was no significant difference in the SH group, which maintained a healthy condition. The total score for knee discomfort in the SH group was significantly lower (P<0.001) at 12 weeks after the start of the regimen than that in the placebo group. Total scores for knee discomfort significantly increased (P=0.015) in the placebo group in a comparison of scores before the start of the regimen and 12 weeks after the start of the regimen, but no significant difference was observed in the SH group, which maintained a healthy condition. Discomfort in the knee when descending stairs at 12 weeks after the start of the regimen was significantly lower in the SH group (P=0.045) than in the placebo group. The score for pain after a longer walk at 12 weeks after the start of the regimen was significantly lower in the SH group (P=0.023) than that in the placebo group. There were no significant differences between the groups with regard to the condition of the knees when bent and extended; however, scores significantly increased in the placebo group (P=0.038) at 12 weeks after the start of the regimen in comparison to the time-point before the start of the regimen. The SH group maintained a healthy condition and exhibited no significant differences. There were no significant differences between the groups with regard to the condition of the knees upon waking, climbing stairs, going to bed and jogging an increased distance or duration compared with the average of the individual.

### Locomotive syndrome risk test

There were no significant differences between the placebo and SH groups, and no significant differences between before and after 12 weeks of the start of the supplement regimen (Table III).

### One-leg standing time with eyes open

There were no significant differences between the placebo and SH groups, and no significant differences between before and after 12 weeks of the start of the supplement regimen (Table III).

### Blood test

There were no significant differences between the placebo and SH groups, and no significant differences between before and after 12 weeks of the start of the supplement regimen (Table IV). No adverse events were caused by the intake of SH during the trial.

## Discussion

The present study was a randomized, double-blinded, placebo-controlled parallel-group study that assessed the effectiveness and safety of the test supplement (SH) on knee discomfort when taken for a period of 12 weeks. Subjects were middle- and old-aged healthy individuals, who had an exercise routine that mostly included jogging and were able to continue with their normal training whilst feeling discomfort in their knees. Subjects with severe gonarthrosis, namely those who scored grade ≥2 based on the KL classification, an indicator of the severity of knee OA, were excluded.

A study evaluating the use of oral SH for individuals with knee OA have reported using paracetamol (an analgesic) as a positive control (17). In addition, crystalline cellulose, dextrin, fatty acid sugar esters and cornstarch have been used as placebo controls (16,23,35). These placebo controls have been reported to have no influence on the knee and no safety concerns. The present study adopted dextrin as a common placebo control in a food supplement study. As it is not a positive control, it could not be compared with medicinal products; however, it was considered to be an appropriate control, as the participants of the present study were considered to be healthy and did not require the administration of medicinal products.

Under the KL classification, a significant association is usually recognized between the severity of gonarthrosis and the JKOM scores, but the degree of such association declines when the severity decreases in relatively mild cases, such as those of grade 1(36). Therefore, as the participants of the present study were graded as ≤1, the association with JKOM scores was reduced and the effect of oral SH was hypothesized to be lower. However, the total points for each VAS used in the present study (pain, stiffness and discomfort) demonstrated that the knee discomfort of the subjects were significantly relieved by oral intake of SH, and this highlights the potential improvement function of SH.

Furthermore, oral intake of SH also alleviated discomfort in the knees when descending stairs and pain in the knees when walking for a longer distance or duration than normal. It is hypothesized that discomfort in the knees when descending stairs was easy to recognize as a subjective symptom, as the body's center of gravity is accelerated downward with the downward motion of the body, which puts a particularly heavy burden on the knee joints (37).

A grade of ≥2 in the KL classification usually increases the rate of knee deformity, articular crepitus and decreased range of motion of the knee (38). Previous SH intake studies on OA have had an 8-week to 12-month duration (23,25,32). The effectiveness of oral SH has been reported to take 12 months in serious cases of grades 2 and 3, demonstrated through an improvement in JKOM scores for elderly subjects aged ≤70 years (23). Conversely, similar previous studies in individuals with mild knee pain or discomfort had a 12-week study period (25,32). In a study of participants with knee OA with an 8-week duration of SH supplementation, a stratified analysis of pain scores of ≥10 determined significant relief of knee pain and discomfort in the SH group compared with the placebo-treated group, but the overall analysis failed to confirm a clear efficacy (16). It was hypothesized that long-term intake is required in KL grade ≥2 with knee OA and 12 weeks of intake is effective in relatively healthy study participants. In the present trial, if individuals with a KL classification of grade ≥2 had been examined, the functionality of SH may have been demonstrated more clearly, even in the locomotive syndrome risk test. However, it is worth noting that a subjective assessment, such as VAS, demonstrated that oral SH alleviates pain and discomfort for healthy individuals who are classified as mild cases.

SH binds to toll-like receptor-4 (TLR-4), present on the mucosal epithelium of the intestine, and suppresses inflammation by promoting the expression of SOCS3 and the production of the regulatory cytokine IL-10 (25,39). The results of the present trial suggested that SH increased the levels of IL-10 (although not statistically significant) as well as an ameliorative effect on knee joint pain, stiffness and discomfort; therefore, this could have possibly involved an anti-inflammatory effect mediated by TLR-4(40). As there is no genetic variation in TLR4 due to ethnicity, the potential function of SH intake on the knee via TLR-4-mediated signal transmission may be extrapolated to other ethnicities, which should be investigated further in the future (41).

Dietary supplements that have been reported to be useful for the improvement of knee discomfort include muco-polysaccharides such as chondroitin, glucosamine and SH. Studies have reported the improvement of knee discomfort using 350 mg/day chondroitin sulfate and 1,500 mg/day glucosamine with healthy adults (42,43). In contrast, reports on the effectiveness of different SH doses range from 48-240 mg/day, which are relatively easy doses to maintain (17,25,44). The present study demonstrated the effect of SH on the knees at a dose of 111 mg/day, which is within the effective range of SH reported in the aforementioned studies, thereby supporting the results of previous research.

An increase in the incidence of OA and a decrease in the amount of endogenous SH are expected in the future due to aging, even for healthy runners such as the subjects of the present study (45,46). Knee OA in particular is the most frequent cause of knee pain in the elderly in Japan (47). This is due to the structural characteristics of the knee joint, which make it inherently unstable, and the biomechanical environment being prone to large loads, making it one of the joints most susceptible to sports injuries and knee OA (47). For middle-aged and elderly individuals who have adopted physical activities centered around running as part of their daily routine, such as the subjects in the present study, training whilst experiencing knee pain or discomfort can further increase the risk of worsening knee pain or discomfort. Therefore, considering the beneficial effect on knee joint pain from the oral intake of SH reported (35,48), effectively utilizing SH as a supplement aimed at alleviating discomfort and suppressing inflammation may contribute to enhanced quality of life for middle-aged and elderly runners who are at risk of such issues.

A limitation of the present study is the lack of objective indicators for objective comparisons, such as before-and-after comparisons of X-ray images, were not performed. To minimize the burden on the study participants, images were taken only once during the study period and were used to select subjects for analysis. Future studies that include objective indices such as imaging are needed.

To conclude, the present study demonstrated that continuous oral intake of SH (111 mg/day) for 12 weeks reduced total VAS scores for pain, stiffness and discomfort in the knees of healthy adults with a KL grade of ≤1, particularly with regard to alleviating discomfort when descending stairs and pain in the knees after walking longer than normal. Furthermore, SH was demonstrated to be safe for use as a functional food with no adverse effects based on blood tests measuring levels of IL-10, AST, ALT, γGTP, LD, CK and CRP. Therefore, the continued intake of SH could potentially contribute to knee health and function. Future studies should investigate whether a synergistic effect can be obtained by combining SH with glucosamine and chondroitin.

## Figures and Tables

**Table I:** Background characteristics of the subjects in the placebo and sodium hyaluronate groups.

Characteristic	Placebo (n=29)	SH (n=27)	P-value
Sex			
Male	23	16	0.103
Female	6	11	
Age, years	61.50±1.59	58.50±1.81	0.219
Height, cm	167.00±1.17	166.00±1.46	0.509
Body weight, kg	60.80±1.71	61.00±2.28	0.966
BMI, kg/m^2^	21.70±0.44	21.90±0.59	0.742

Values are expressed as n or the mean ± standard error. P-values were determined using the Chi-squared test for the number of participants and unpaired t-tests for all other items. SH, sodium hyaluronate; BMI, body mass index.

**Table II:** Changes from baseline on visual analog scales for knee pain, stiffness and discomfort during certain activities after 6 and 12 weeks of ingestion of placebo or SH.

	6 weeks		12 weeks	
Item	Placebo (n=15)	SH (n=16)	Placebo (n=15)	SH (n=16)
Total				
Pain	-0.11±0.06	0.09±0.08^a^	0.44±0.18^b^	-0.18±0.10^a^
Stiffness	0.18±0.08	0.21±0.10	0.21±0.08^c^	-0.08±0.09^d^
Discomfort	0.13±0.10	0.09±0.08	0.33±0.13^c^	-0.17±0.09^a^
Upon waking				
Pain	-0.27±0.25	0.19±0.14	0.18±0.42	0.07±0.26
Stiffness	0.38±0.16	0.31±0.15	0.19±0.35	0.16±0.25
Discomfort	0.29±0.25	0.16±0.20	0.83±0.63	0.04±0.25
Climbing stairs				
Pain	-0.19±0.15	0.22±0.22	0.30±0.24	0.03±0.25
Stiffness	0.44±1.30	0.17±0.18	0.37±0.18	-0.08±0.31
Discomfort	0.34±1.35	0.04±0.20	0.38±0.35	-0.25±0.30
Descending stairs				
Pain	-0.10±0.10	0.10±1.25	0.71±0.51	-0.29±0.23
Stiffness	0.02±0.09	0.17±0.16	0.29±0.16	-0.22±0.23
Discomfort	-0.16±0.17	0.02±0.17	0.34±0.27	-0.44±0.25^d^
Going to bed				
Pain	0.01±0.06	0.11±0.11	0.25±0.16	0.06±0.12
Stiffness	0.05±0.12	0.15±0.13	0.15±0.11	0.03±0.09
Discomfort	-0.06±0.07	0.11±0.16	0.09±0.12	-0.03±0.08
Bending and stretching knees				
Pain	-0.05±0.22	0.30±0.47	0.62±0.29^c^	0.03±0.17
Stiffness	0.45±0.27	0.64±1.24	0.35±0.14	0.03±0.18
Discomfort	0.26±0.19	0.25±0.29	0.37±0.31	-0.19±0.24
Walking for a longer distance/time compared with usual				
Pain	-0.13±0.30	-0.03±0.23	0.53±0.33	-0.38±0.20^d^
Stiffness	0.13±0.31	0.07±0.25	0.04±0.21	-0.21±0.17
Discomfort	0.23±0.31	0.06±0.25	0.27±0.35	-0.21±0.19
Jogging for a longer distance/time compared with usual				
Pain	-0.07±0.24	-0.26±0.33	0.44±0.42	-0.85±0.45
Stiffness	-0.31±0.22	-0.03±0.35	0.12±0.33	-0.25±0.31
Discomfort	-0.01±0.22	0.02±0.35	0.01±0.32	-0.11±0.32

Data are presented as the mean ± standard error.

^a^P<0.001 SH vs. placebo group;

^b^P<0.001 vs. week 0;

^c^P<0.05 vs. week 0;

^d^P<0.05 SH vs. placebo group. P-values were determined using two-way mixed ANCOVA with a Sidak post-hoc test. SH, sodium hyaluronate; VAS, visual analog scale.

**Table III:** Results of the locomotive syndrome risk test and one-leg standing time with eyes open test at baseline and at 12 weeks after starting treatment.

	0 weeks		12 weeks	
Test	Placebo (n=15)	SH (n=16)	Placebo (n=15)	SH (n=16)
Stand-up test score	3.67±0.45	3.38±0.27	3.20±0.39	2.63±0.29
Two-step test, 2 step value^a^	1.56±0.03	1.58±0.04	1.63±0.03	1.63±0.05
GLFS-25 score	7.47±1.90	6.25±1.27	5.21±1.09	5.50±1.08
One-leg standing time with eyes open test, sec				
Right leg	111±4.48	101±10.0	116±4.00	98.6±9.83
Left leg	100±8.76	96.3±9.37	108±6.72	107±8.31

Data are presented as the mean ± standard error.

^a^2 step value=2 step length (cm)/height (cm). Statistical analysis of the data for the stand-up test and GLFS-25 was performed using the Wilcoxon signed-rank test with Bonferroni correction for 0 vs. 12 weeks comparisons and the Mann-Whitney U-test with Bonferroni correction for placebo vs. SH comparisons; statistical analysis of the data for the two-step test and one-leg standing time with eyes open test was performed using two-way mixed ANOVA and Sidak post-hoc test. SH, sodium hyaluronate; GLFS-25, 25-question geriatric locomotive function scale.

**Table IV:** Results of blood tests at baseline and at 12 weeks after the start of the supplement regimen.

		0 weeks		12 weeks	
Parameter	Normal reference values	Placebo (n=15)	SH (n=16)	Placebo (n=15)	SH (n=16)
AST, U/l	10-40	32.5±3.37	28.3±1.58	31.2±3.14	26.3±1.66
ALT, U/l	5-45	26.9±3.33	22.7±2.15	26.7±2.93	23.0±2.59
LDH, U/l	120-220	202±8.36	216±8.05	214±9.33	213±9.81
γGT, U/l	Male: 0-80	47.5±10.1	43.3±9.17	51.6±15.8	49.7±16.8
	Female: 0-30				
CK, U/l	Male: 60-250	213±32.4	283±66.9	381±163.8	164±18.3
	Female: 50-170				
CRP, mg/dl	≤0.35	0.06±0.01	0.06±0.02	0.12±0.03	0.06±0.01
IL-10, pg/ml	<8	5.67±1.24	5.19±0.88	5.76±0.99	5.68±0.79

Data are presented as the mean ± standard error. Statistical analysis of the data was performed using two-way mixed ANOVA with a Sidak post-hoc test. SH, sodium hyaluronate; AST, aspartate aminotransferase; ALT, alanine transaminase; LDH, lactate dehydrogenase; γGT, γ-glutamyl transferase; CK, creatine kinase; CRP, C-reactive protein; IL-10, interleukin-10.

## Data Availability

The datasets used and/or analyzed during the current study are available from the corresponding author on reasonable request.
